# Effect of Helium on Dispersoid Evolution under Self-Ion Irradiation in A Dual-Phase 12Cr Oxide-Dispersion-Strengthened Alloy

**DOI:** 10.3390/ma12203343

**Published:** 2019-10-14

**Authors:** Hyosim Kim, Tianyao Wang, Jonathan G. Gigax, Shigeharu Ukai, Frank A. Garner, Lin Shao

**Affiliations:** 1 Los Alamos National Laboratory, P.O. Box 1663, Los Alamos, NM 87544, USA; jgigax@lanl.gov; 2 Department of Nuclear Engineering, Texas A&M University, College Station, TX 77843-3128, USA; wty19920822@tamu.edu (T.W.); frank.garner@dslextreme.com (F.A.G.); 3 Materials Science and Engineering, Faculty of Engineering, Hokkaido University, N13, W-8, Kita-ku, Sapporo, Hokkaido 060-8628, Japan; s-ukai@eng.hokudai.ac.jp

**Keywords:** oxide-dispersion-strengthened (ODS), ion irradiation, He implantation, dual-phase, ferritic-martensitic, self-ion

## Abstract

As one candidate alloy for future Generation IV and fusion reactors, a dual-phase 12Cr oxide-dispersion-strengthened (ODS) alloy was developed for high temperature strength and creep resistance and has shown good void swelling resistance under high damage self-ion irradiation at high temperature. However, the effect of helium and its combination with radiation damage on oxide dispersoid stability needs to be investigated. In this study, 120 keV energy helium was preloaded into specimens at doses of 1 × 10^15^ and 1 × 10^16^ ions/cm^2^ at room temperature, and 3.5 MeV Fe self-ions were sequentially implanted to reach 100 peak displacement-per-atom at 475 °C. He implantation alone in the control sample did not affect the dispersoid morphology. After Fe ion irradiation, a dramatic increase in density of coherent oxide dispersoids was observed at low He dose, but no such increase was observed at high He dose. The study suggests that helium bubbles act as sinks for nucleation of coherent oxide dispersoids, but dispersoid growth may become difficult if too many sinks are introduced, suggesting that a critical mass of trapping is required for stable dispersoid growth.

## 1. Introduction

Oxide-dispersion-strengthened (ODS) alloys are one class of promising candidate alloys for Gen IV and fusion reactors due to their superior high temperature strength and creep resistance [1,2,3,4,5,6,7]. Among various ODS alloys developed worldwide, a dual-phase ferritic/martensitic (F/M) 12Cr ODS alloy has shown good high-temperature oxidation resistance and corrosion resistance [3]. This alloy has specifically controlled excess oxygen and titanium contents to control residual alpha ferrite volume for superior creep rupture strength [1]. A high level of tempered martensite (TM) volume in the matrix contributes to good void swelling resistance [4]. Recent studies have shown good grain stability at 800 displacement-per-atom (dpa) after 600 °C self-ion irradiation, and good swelling resistance (less than 2% and 0.06% at 475 °C for the ferrite and TM phases, respectively) [8,9]. These studies also show that oxide dispersoid size changes saturate with increasing dose. It has been suggested that the increased dispersoid density upon irradiation may improve the swelling resistance by providing more recombination sites for point defects [10,11]. A previous study on the same alloy using the same irradiation condition employed in this study showed that the dispersoid density increased in both ferrite and TM phases due to ballistic dissolution by irradiation and thermodynamic homogenous nucleation at high temperature [10,12]. Due to these properties, the 12Cr ODS alloy is promising for applications in future reactor designs where the operation conditions are more severe with higher damage levels and higher temperatures [13].

However, to deploy this alloy into a fast reactor environment, the effect of helium addition produced by (n, α) transmutation and subsequent He embrittlement needs to be further studied [14]. There have been a few He implantation studies on various ODS alloys [11,15,16,17,18]. Yamamoto et al. observed small-size He bubbles trapped on YTiO clusters during simultaneous neutron and He irradiation of MA957 [15]. Edmondson et al. also reported similar results on 14YWT after He implantation alone at high temperature [16]. Yutani et al. investigated the He effect on ODS alloys using single and dual-ion irradiations at high temperature and reported that smaller nano-sized particles can induce a high density of small He bubbles and thereby reduce swelling [11]. Heintze et al. showed that radiation behaviors of ODS alloys are different for single, dual, and sequential He and Fe ion irradiation [17]. Lu et al. reported that dispersoid densities increase after sequential He and Fe ion irradiation of 9Cr ODS alloys [18].

Although many He ion implantation studies have been conducted, there is still a need to further investigate the He effect, particularly in the 12Cr ODS alloy. First, the majority of previous studies focused on swelling, with little attention on dispersoid stability. Dispersoid morphology changes play a significant role in influencing creep resistance, which was one of the main motivations for introducing ODS alloys into nuclear applications. Second, there is no previous report on the He effect on the TM phase of ODS alloys and is unclear if the effect is similar to that reported in ferritic and austenitic ODS alloys. The TM phase has demonstrated much better swelling resistance than the ferrite phase. Thereby TM phase-dominated ODS alloys are very attractive to further improve swelling resistance. 

## 2. Materials and Experimental Procedure

The dual-phase 12Cr ODS alloy was fabricated using a mechanical alloying (MA) process in Hokkaido University (Sapporo, Japan). The MA processed powders were consolidated using a spark plasma sintering method at 1100 °C and were hot-rolled afterward. The final steps involved normalization at 1050 °C for 1 h and tempering at 800 °C for 1 h. More details on the fabrication process can be found in [3]. The chemical composition of the alloy is provided in Table 1. The 12Cr ODS alloy was cut into 3 mm × 6 mm × 1.5 mm pieces. Samples were then mechanically polished by using SiC paper up to p-4000 fine grit, and further polished with 0.25 µm diamond suspension and 0.04 µm silica suspension to remove surface deformation. The final sample thickness was ~0.7 mm. 

**Figure 1:**
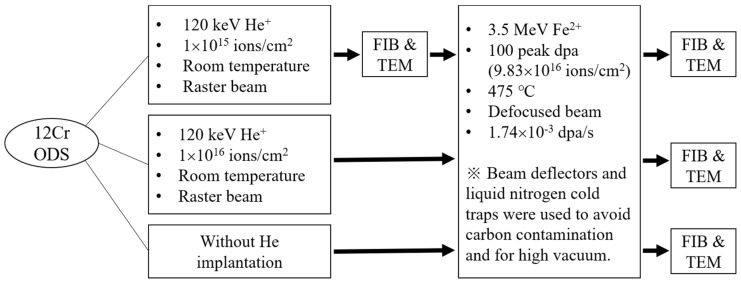
Diagram of experimental steps.

The experiment design is presented in Figure 1. Two pristine specimens were irradiated using a rastered 120 keV He^+^ ion beam to a dose of 1 × 10^15^ and 1 × 10^16^ ions/cm^2^, respectively, both at room temperature. The specimens were then irradiated using a 3.5 MeV Fe^2+^ defocused ion beam to reach 100 peak dpa at 475 °C using a 1.7 MV tandem accelerator. The average dpa rate was 1.74 × 10^−3^ dpa/s for the Fe ion beam and the ion fluence yielding 100 peak dpa was 9.83 × 10^16^ ions/cm^2^. Note that the Fe irradiation utilized a multiple beam deflection technique to filter out carbon and other contaminants [19,20,21,22]. The target chamber vacuum was at 4.0 × 10^−8^–6.0 × 10^−8^ torr during the irradiation. Additionally, liquid nitrogen cold trapping in the target chamber was applied [19,20,21,22]. For He implantation, a raster beam was used to guarantee beam uniformity over a large irradiation area. The rastering effect was not a concern for He implantation since the implantation was used only to introduce He bubbles. For the Fe irradiation, however, a static defocused beam was used [23]. Our study further included control samples, some irradiated by He ions only, and others by Fe ions only. 

All samples were characterized using a Tescan Lyra-3 transmission electron microscope (TEM). The focused ion beam (FIB) lift-out technique was used to prepare TEM lamella specimens [24], using a FEI Tecnai G2 F20 Super-Twin (FEI, Hillsboro, OR, USA). The TEM specimen thickness was measured using electron energy loss spectroscopy (EELS) on a FEI Tecnai F20. Bright-field (BF) and weak-beam dark-field (WBDF) imaging techniques were used to characterize dispersoid coherency and distribution. High-resolution TEM (HRTEM) and fast Fourier transform (FFT) were used to confirm the crystal structure of the dispersoids. The WBDF imaging is a diffraction-contrast imaging using a weakly excited beam. It is widely used for imaging sub-nano-size features because of better accuracy on position than normal dark-field (DF). While the desired g (for this study, g110 of matrix) is on the optical axis and used for a regular DF, the sample is tilted to make the 3g diffraction pattern brightest to make s_g_ (excitation error) large. This allows diffracting planes to bend locally back into the Bragg-diffracting orientation to give more intensity in the DF image [25,26,27].

The average dispersoid diameter was calculated by averaging diameters measured one-by-one from BF and DF micrographs. Dispersoids were counted only when they were present in both BF and DF images. The dispersoid density was calculated by dividing the number of counted coherent dispersoids with (area × specimen thickness) in areas where dispersoids were counted.

**Figure 2:**
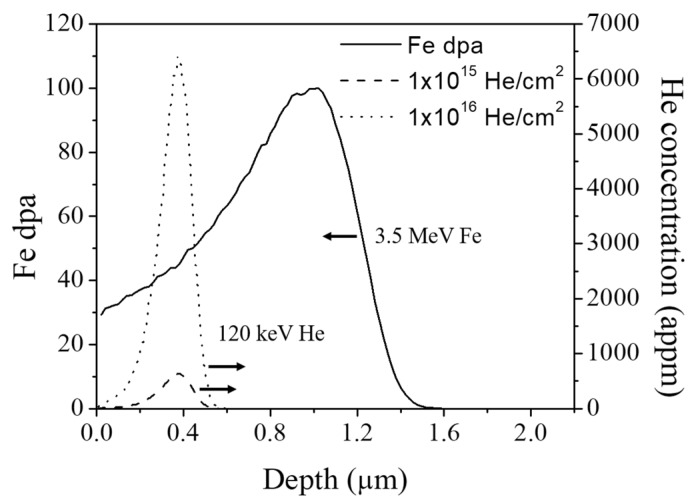
Stopping and Range of Ions in Matter (SRIM) calculations of damage profiles resulting from 3.5 MeV Fe ion irradiation and the He distributions resulting from 120 keV He ion implantation.

The Stopping and Range of Ions in Matter (SRIM) 2013 code was used to calculate damage profiles and implant distributions. The dpa calculation used the Kinchin–Pease mode and the Fe displacement threshold energy was chosen to be 40 eV [28,29]. Figure 2 shows the SRIM calculation of the 120 keV He implant profiles for doses of 1 × 10^15^ and 1 × 10^16^ ions/cm^2^ and the dpa profile for 3.5 MeV Fe 100 peak dpa irradiation. The 3.5 MeV Fe beam penetrates to a maximum depth of 1.5 µm below the incident surface, and the dpa peak is ~1 µm deep. In the case of 120 keV He, the maximum penetration depth is approximately 0.5 µm from the surface. The dashed line refers to the 1 × 10^15^ He/cm^2^ case with a He peak at ~600 appm, while the dotted line refers to the 1 × 10^16^ He/cm^2^ case with a He peak at ~6000 appm. The He/dpa ratio at the He peak depth is 14.9 appm/dpa for the 1 ×10^15^ He/cm^2^ and 148.8 He/dpa for the 1 × 10^16^ He/cm^2^. An energy of 120 keV is selected for He implantation to avoid the free surface effect and to minimize the injected interstitial effect during subsequent Fe ion irradiation [30]. Since void swelling by 3.5 MeV ion irradiation is usually maximized in the front half of the projected range [30], it is ideal to introduce He into the depth region of 300–400 nm. Note that the average local dpa for this region is about 43 dpa. 

## 3. Results

### 3.1. He Implantation Only

**Figure 3:**
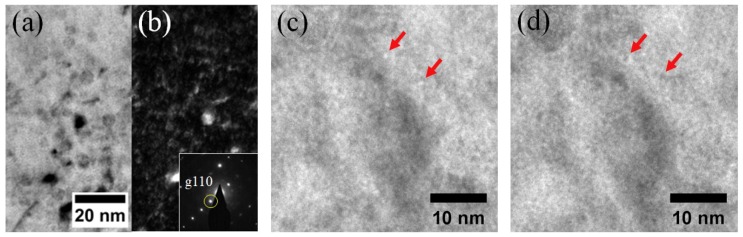
(**a**) Bright-field, (**b**) dark-field, (**c**) under-focused, and (**d**) over-focused TEM images taken from 300–400 nm depth region, after 1 × 10^15^ He ions/cm^2^ implantation. The diffraction pattern used to obtain BF and DF images is superimposed on (**b**) with g110 indexed. He bubbles appear bright and dark in under-focused and over-focused images, respectively.

TEM images were taken from the 350 nm depth region where the He ion peak is located to see the effect of He implantation on oxide dispersoid size and density. Figure 3a,b shows BF and DF images of the He implanted sample using (g, 3 g) condition with the g110 direction excited as indicated by the inset of the diffraction image shown in Figure 3b [24]. The diffraction pattern was obtained from the same grain where the BF and DF images were taken. A total of 33 coherent oxide dispersoids were counted in the 300 to 400 nm depth region. The oxide dispersoids have dark contrast in the BF image regardless of coherency, while only coherent dispersoids appear bright in DF imaging. Other features such as small dislocation loops or dislocation lines can also appear bright if they are aligned with g110. However, dislocation lines and dislocation loops are distinguishable from oxide dispersoids in their shape and contrast under the BF imaging mode. For example, a narrow and long dark contrast line is a dislocation line as shown in the bottom left corner of Figure 3a, which is clearly distinguishable from dispersoids. Small dislocation loops (≤4 nm) can be difficult to separate from small dispersoids. In this study, three factors were considered when distinguishing loops. First, if the shape is not circular, it was not counted as a dispersoid. Second, if it appears as a black dot rather than as a slightly dark contrast, it is considered as a loop or dislocation core. Small dispersoids mostly exhibit a coherent relationship with matrix, yielding slightly darker contrast than the matrix. Third, if the feature has a black-white contrast, it is a loop lying close to the foil surface [27]. By comparing BF and DF images, coherent and incoherent dispersoids can be counted separately. Note that only the TM phase was analyzed in this study for two reasons. First, the TM phase accounts for 80% of the volume of the alloy. Second, the ferrite phase shows a dramatic dispersoid density change even without He implantation (as shown in the previous study reported in [10]). In the present study only coherent dispersoids were counted because newly nucleated oxide dispersoids are prone to take a coherent relationship with the matrix to achieve lower surface energy [31,32].

Figure 3c,d shows TEM BF under-focused and over-focused images, respectively, taken from the 300 to 400 nm depth region in the 1 × 10^15^ He ions/cm^2^ implanted specimen. In the under-focused image, He bubbles appear white, while they appear dark in the over-focused image, as indicated by red arrows. The He bubble size was measured to be less than 1 nm diameter at this depth. Due to the small size, it is challenging to obtain size and density from the TEM image. The size and density of oxide dispersoids were counted from this region, and the results are discussed in Section 3.4.

**Figure 4:**
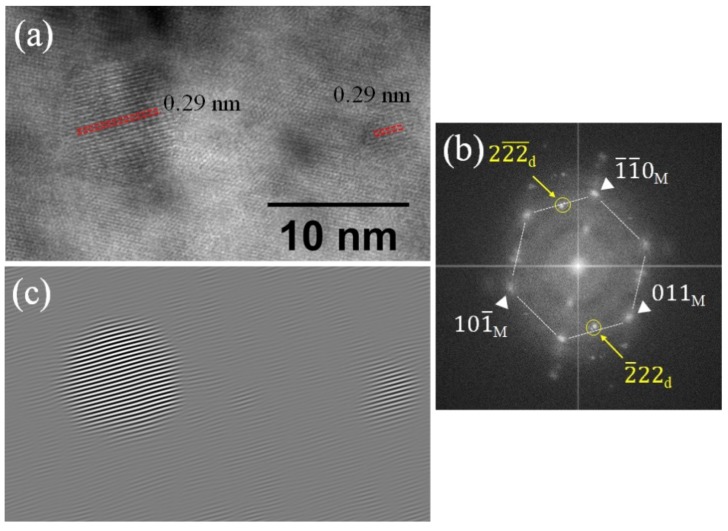
(**a**) High-resolution TEM (HRTEM) of two oxide dispersoids, (**b**) fast Fourier transform (FFT) patterns from the HRTEM, and (**c**) inverse FFT image of dispersoids using FFT patterns in yellow circles. The *d*-spacings measured from HRTEM and FFT patterns are 0.29 nm, which agree with (222) of pyrochlore Y_2_Ti_2_O_7_. The scale bar in (a) applies to (c).

Figure 4a shows a HRTEM image of two different size oxide dispersoids obtained from the He implanted region. The HRTEM image was taken at the $\left[\bar{1}1\bar{1}\right]$ zone axis of the matrix. Figure 4b shows the FFT patterns from the matrix and dispersoids that are indexed separately using white triangles and yellow arrows, respectively. The *d*-spacings of dispersoids measured from HRTEM and FFT patterns are 0.29 nm, which agree with (222) plane spacing of pyrochlore Y_2_Ti_2_O_7_. Previous studies on this alloy also showed that the oxide dispersoids are either orthorhombic Y_2_TiO_5_ or pyrochlore Y_2_Ti_2_O_7_ [8,9]. FFT patterns from the dispersoids (yellow circles in Figure 4b) were used to generate an inverse FFT image as shown in Figure 4c to highlight the dispersoids in Figure 4a.

### 3.2. Fe Irradiation Only

**Figure 5:**
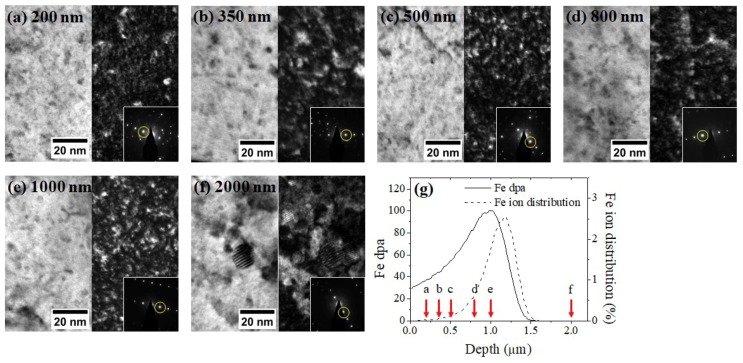
TEM bright-field and dark-field images of a Fe irradiated sample, taken from depths of (**a**) 200, (**b**) 350, (**c**) 500, (**d**) 800, (**e**) 1000, and (**f**) 2000 nm. Diffraction patterns obtained from the same grain are superimposed on each DF image with g110 marked by a yellow circle. (**g**) SRIM Fe damage profile is shown with red arrows indicating the depths of characterization. The dispersoid size decreased and the density increased within the ion range.

Figure 5 shows TEM BF and DF images taken from the Fe-irradiated sample at selected depths. Images were taken from six different depths, including the out-of-ion range region (2000 nm). The 2000 nm region was used as a reference point, since it is free from direct ion bombardment (although it does have a possible thermal annealing effect during the irradiation). The same WBDF condition utilized for Figure 3b imaging was used for consistency. Figure 5g shows SRIM damage profile and red arrows are used to show the depths of the TEM characterization. More than 70 coherent oxide dispersoids were counted from each set of micrographs. As shown in Figure 5, large incoherent dispersoids are observed at 2000 nm depth, while only small-size, mostly coherent dispersoids are observed within the ion range (≤1000 nm). 

### 3.3. He Implantation Followed by Fe Irradiation

**Figure 6:**
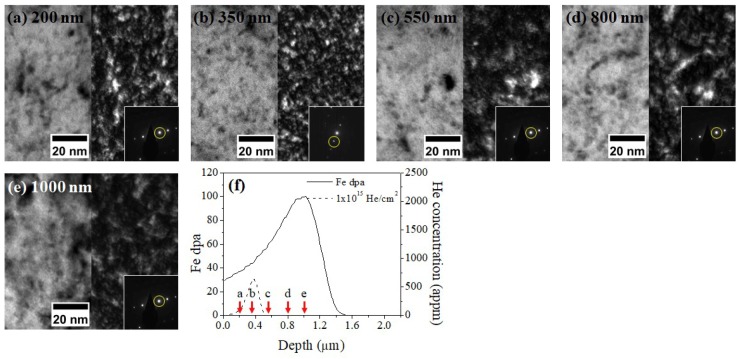
TEM bright-field and dark-field images of the 1 × 10^15^ He preimplanted and Fe irradiated sample at depths of (**a**) 200, (**b**) 350, (**c**) 550, (**d**) 800, and (**e**) 1000 nm. Diffraction patterns obtained from the same grain are superimposed on each DF image with g110 marked by a yellow circle. (**f**) SRIM calculations of Fe, dpa, and He implant distributions are included, with red arrows indicating the depths of characterization. The dispersoid density was greatly increased in (**b**).

Figure 6 shows TEM BF and DF micrograph sets of the 1 × 10^15^ He preimplanted and Fe irradiated sample taken from 200, 350, 550, 800, and 1000 nm depths and SRIM calculations of He implant distribution and Fe damage. The same WBDF condition employed in Figure 5 was used. At a depth of 350 nm, corresponding to the He peak location, the TEM DF image in Figure 6b shows the highest density of coherent dispersoids. This is evidence that nucleation of coherent oxide dispersoids is promoted there. 

**Figure 7:**
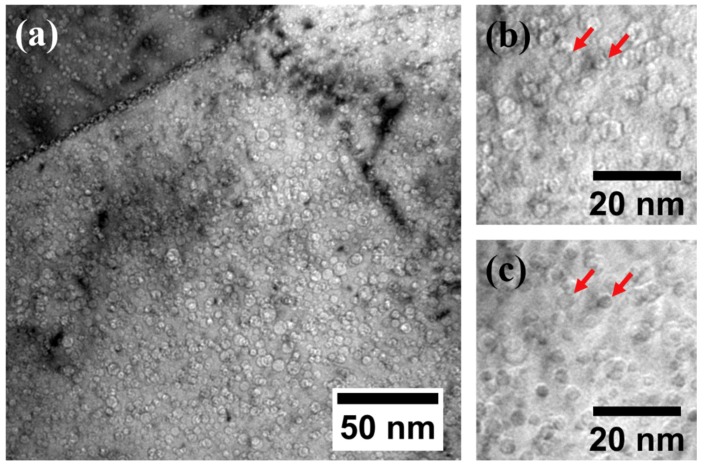
(**a**) Bright-field TEM micrograph, (**b**) under-focused image, and (**c**) over-focused image from the 300–500 nm depth region of the 1 × 10^16^ He preimplanted and Fe irradiated specimen.

Figure 7 shows TEM BF images of 1 × 10^16^ He + Fe irradiated sample taken from 300–500 nm depth region. The average diameter of He bubbles was measured to be 3.4 ± 0.9 nm. A high number of He bubbles were observed on the grain boundaries, as shown in Figure 7a, suggesting a sink effect of the grain boundaries. The He bubble density at this depth was measured to be 1.6 × 10^23^ ± 1.3 × 10^22^ bubbles/m^3^. A He bubble denuded zone was observed near grain boundaries. Figure 7b,c shows under-focused and over-focused images of He bubbles at higher magnification, respectively, taken from the same region. He bubbles appear bright with enhanced edge contrast in under-focus images while they appear dark with a vague boundary in over-focus images. Examples are indicated by the red arrows in Figure 7b,c.

**Figure 8:**
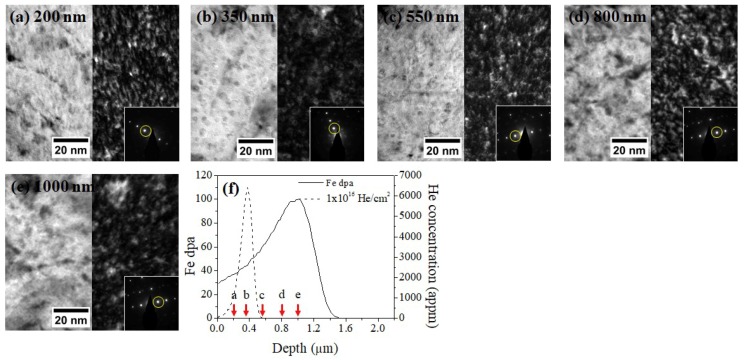
TEM bright-field and dark-field micrographs of the 1 × 10^16^ He preimplanted and Fe irradiated specimen taken from depths of (**a**) 200, (**b**) 350, (**c**) 550, (**d**) 800, and (**e**) 1000 nm. Diffraction patterns obtained from the same grain are superimposed on each DF image with g110 marked by a yellow circle. (**f**) SRIM calculations are provided to show the depths of TEM characterization. A dispersoid density increase was not observed in (**b**).

Figure 8 shows TEM BF and DF micrographs of the 1 × 10^16^ He preimplanted and Fe irradiated sample taken from 200, 350, 550, 800, and 1000 nm depths, along with the SRIM calculations. Unlike the DF image at 350 nm of the 1 × 10^15^ He + Fe specimen, the DF image of the 1 × 10^16^ He + Fe sample does not show many bright features, suggesting that the higher dose of He preimplantation did not lead to more nucleation of oxide dispersoids during Fe irradiation. Further analysis on the size and density of dispersoids is given in Section 3.4.

### 3.4. Oxide Dispersoid Size and Density Comparison

**Figure 9:**
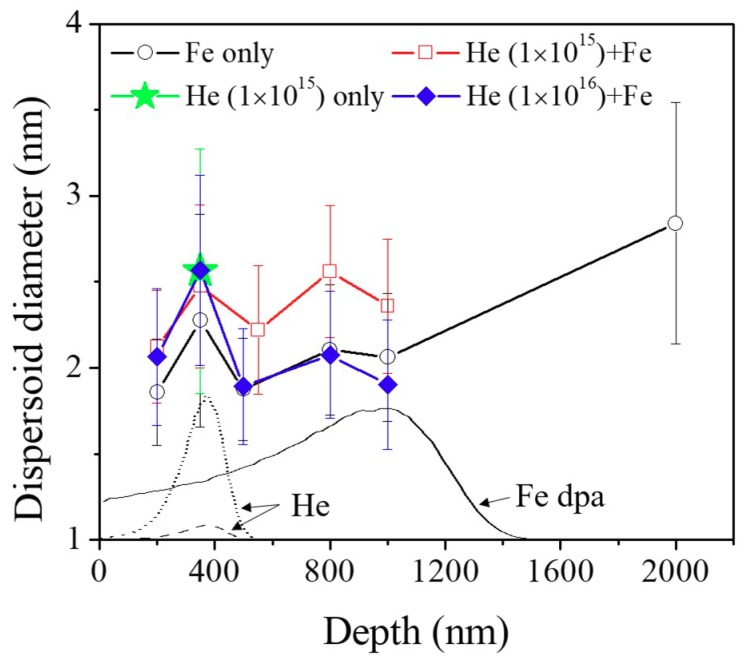
Average dispersoid diameters of Fe irradiated only, He implanted only, and He + Fe irradiated samples as a function of depth. The SRIM calculations are superimposed. The average size decreased within the ion range, but no significant difference was observed between the specimens.

Figure 9 shows the average oxide dispersoid diameters of different irradiation conditions as a function of depth. The superimposed dashed and dotted lines refer to the 1 × 10^15^ and 1 × 10^16^ 120 keV He ion distributions, respectively, and the black solid line shows the Fe damage profile. The Fe irradiation sample (hollow circles) shows that the dispersoid sizes within the ion range are roughly the same considering the error bars. This uniformity can be explained by the fact that oxide dispersoid size is not dependent on local dpa rates as shown in a previous study [24]. Compared with the out-of-ion range region (2000 nm), oxide dispersoid sizes within the ion range are reduced after Fe irradiation due to ballistic dissolution [8,9,24]. 

The size of dispersoids after room temperature 1 × 10^15^ He implantation, indicated by the star in Figure 9, is almost the same as the size of oxide dispersoids at 2000 nm depth, which suggests that He implantation itself does not affect dispersoid morphology. The 1 × 10^15^ He + Fe irradiated sample shows slightly larger dispersoid sizes within the heavy ion range, but this is actually not related to the He preimplantation effect, since the size is still widely distributed at regions >600 nm. Therefore, considering the error bars, we believe it is a statistical fluctuation varying from sample to sample or grain to grain. The 1 × 10^16^ He + Fe irradiated specimen indicated by solid diamonds shows that dispersoid sizes are similar to that of the Fe irradiated specimen. Overall, there is no clear trend that He preimplantation affects dispersoid size after subsequent Fe ion irradiation. 

**Figure 10:**
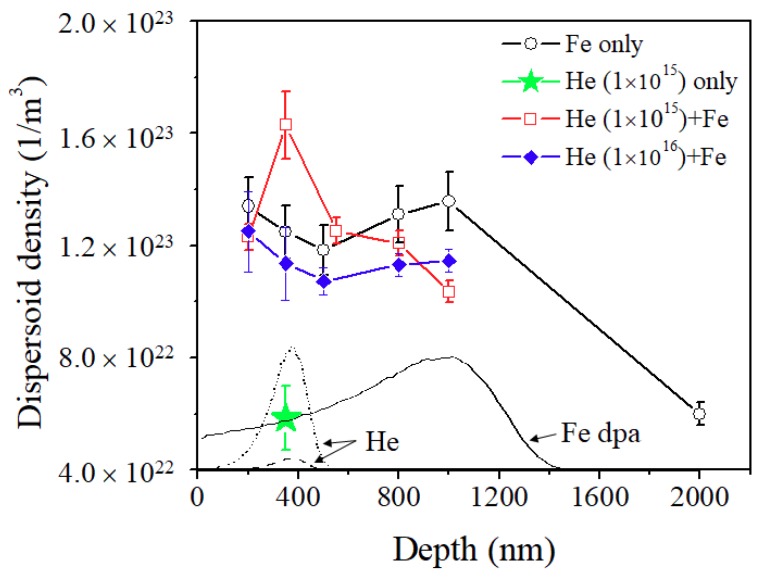
Dispersoid densities of Fe irradiated, He implanted and He + Fe irradiated samples as a function of depth. The He ion profiles and Fe damage curve are superimposed. The densities increased within the ion range for all cases, and the highest density was observed after the 1 × 10^15^ He + Fe irradiation.

Figure 10 shows the dispersoid densities as a function of depth, superimposed with the SRIM calculations. The He implanted-only sample (indicated by stars) shows a density similar to that of the out-of-ion-range of the Fe irradiated sample (2000 nm), which implies that He implantation alone does not affect the dispersoid density, at least when implanted at room temperature at 120 keV energy.

The coherent dispersoid densities, however, are dramatically increased after Fe irradiation as indicated by circles, and there is a factor of two density increase at 350 nm depth, in comparison with the point at 2000 nm. On the other hand, the 1 × 10^15^ He preimplanted + Fe irradiated sample (indicated by squares) shows the highest coherent dispersoid density at the He ion peak location (350 nm). The enhancement is a factor of 2.7 in comparison with the 2000 nm reference point. The coherent oxide dispersoid size and density for each case at 350 nm depth and 2000 nm depth are summarized in Table 2.

For the 1 × 10^16^ He preimplanted + Fe irradiated specimen (indicated by solid diamonds), the densities of dispersoid are systematically lower than those of the Fe irradiated sample within the ion range (≤1000 nm). This suggests that the large-size He bubbles do not affect dispersoid nucleation under Fe ion irradiation, and there is a possibility that the large bubbles even suppress the nucleation process. It appears that small-size He bubbles in the matrix assist coherent oxide dispersoid nucleation during Fe irradiation, resulting in a higher density of coherent dispersoids at the He peak region. However, this effect occurs only when the He bubble size is small. 

**Figure 11:**
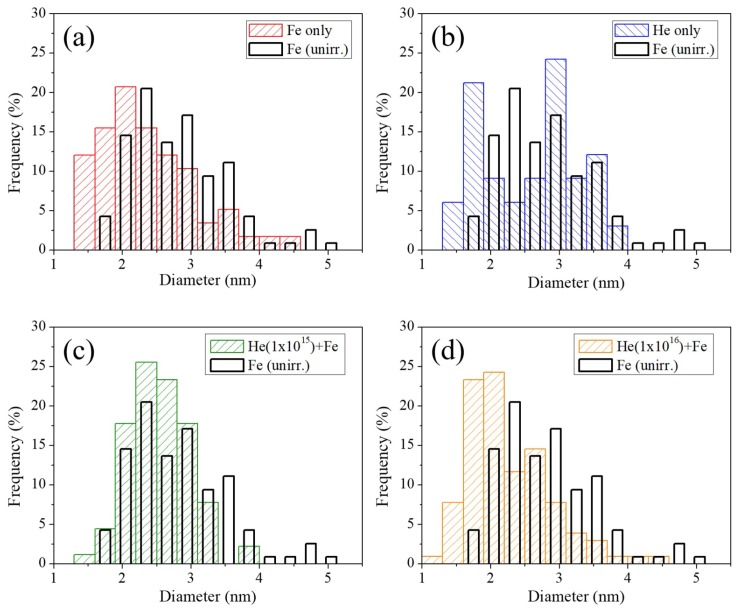
Dispersoid size distributions of (**a**) Fe irradiated, (**b**) He implanted-only, (**c**) He (1 × 10^15^) + Fe, and (**d**) He (1 × 10^16^) + Fe irradiated samples from the 350 nm depth. Black lines superimposed on each figure are the dispersoid size distribution at 2000 nm depth of Fe irradiated specimen.

Figure 11 shows dispersoid size distributions of Fe irradiated, He implanted, 1 × 10^15^ He + Fe irradiated, and 1 × 10^16^ He + Fe irradiated specimens taken from the 350 nm depth region. Black solid lines superimposed on each figure are the dispersoid size distribution of the 2000 nm depth region from the Fe irradiated sample for comparison. With an exception of the He implanted-only sample, the size distributions follow a gaussian distribution. Figure 11a shows that the dispersoid size of the 350 nm depth of the Fe irradiated specimen is skewed toward a lower depth, due to ballistic dissolution. Figure 11b suggests a bi-modal distribution after 1 × 10^15^ He implantation, with a high density of dispersoids of both ~3 nm and <2 nm observed. 

The 1 × 10^15^ He + Fe sample shows a size distribution similar to the out-of-range reference point, but with fewer large dispersoids and a higher frequency of small dispersoids. This means that large dispersoids dissolved under Fe irradiation, and small coherent dispersoids nucleated. Figure 11d shows the size distribution of 1 × 10^16^ He + Fe specimen, and appears very similar to that of Figure 11a. This means that a high dose of He preimplantation at room temperature does not affect the dispersoid size or size distribution. As only coherent dispersoids were counted in TM phase, the dispersoid size distribution remains in the same range (1.5–5.5 nm diameter) for all cases.

## 4. Discussion

As its migration energy is low at 0.078 eV, helium can migrate easily and become trapped at defects such as vacancies, dislocations, grain boundaries, and precipitate surfaces [33]. When He is trapped by a vacancy, He-vacancy clusters are formed in the matrix [34,35,36,37,38], and these He-vacancy clusters can attract oxygen due to their high vacancy-oxygen affinity. According to a previous density functional theory (DFT) study on the vacancy mechanism of high oxygen solubility and nucleation of stable oxygen enriched clusters in Fe, oxygen in an interstitial position shows a high affinity for vacancies due to a weak bonding with the Fe matrix [39]. This O-vacancy mechanism further enables the nucleation of O-enriched nanoclusters, and it further attracts solutes like Ti and Y with high oxygen affinities. When trapped elements reach certain concentrations, dispersoids are nucleated. In a similar mechanism, when oxide dispersoids and He bubbles are present in the matrix, dispersoids will dissolve under irradiation through ballistic dissolution. Then, oxygen from dispersoids and matrix will form O-enriched nanoclusters at He-vacancy clusters which further attracts Ti and Y to form oxide dispersoids. We believe that an increase of coherent dispersoid density in the 1 × 10^15^ He + Fe irradiation case is due to this mechanism. 

It is unclear at this stage why a higher He ion fluence at 1 × 10^16^ ions/cm^2^ does not lead to further increases in dispersoid density. We speculate that at much higher He fluence, the number density of small cavities or He-vacancy clusters is larger. Thus the amount of O/Ti/Y trapped per cavity may be reduced significantly. If there is a critical trapping mass required to reach a stable dispersoid, in a way similar to formation of stable void nuclei, dispersoid growth might be difficult if the trap density is too large.

The observed opposite trend of He effect on dispersoids appears to be quite similar to the often-observed He effect on swelling. In previous helium co-implantation studies on various non-ODS alloys, it has been reported that helium does not always increase swelling [40,41,42,43]. Void swelling increased for low He/dpa levels and decreased for high He/dpa levels. Helium promotes cavity nucleation. At low He/dpa levels, cavities are defect sinks for vacancies, and interstitial loops/dislocations are biased sinks for interstitials. However, if the cavity density is too high at high He/dpa levels, cavities become the dominant defect sinks. Since cavities are thought to be neutral sinks for both interstitials and vacancies, defect recombination is promoted instead of biased vacancy trapping. This leads to limited and saturated void growth [43].

## 5. Conclusions

A dual-phase F/M 12Cr ODS alloy was preloaded with He atoms by using 120 keV He^+^ ions at room temperature at two different fluences of 1 × 10^15^ and 1 × 10^16^ ions/cm^2^. He was first implanted and followed by irradiation with 3.5 MeV Fe^2+^ ion at 475 ℃ to 100 peak dpa. The He preimplantation effect on the oxide dispersoids in the TM phase was investigated for the first time in this study. The coherent oxide dispersoids’ size and density were characterized as a function of depth. The 1 × 10^15^ ions/cm^2^ He implantation itself did not change dispersoid sizes. However, the dispersoid density was increased after sequential He + Fe ion irradiation for the He dose of 1 × 10^15^ ions/cm^2^. On the other hand, such enhancement was not observed for the higher He dose of 1 × 10^16^ ions/cm^2^, suggesting that additional bubble and vacancy cluster trapping may dilute local solute concentrations, which makes the initial nucleation stage difficult. 

## Figures and Tables

**Table 1:** Chemical composition of as-received dual-phase 12Cr oxide-dispersion-strengthened (ODS) alloy (wt %).

Fe	Cr	W	Ni	Ti	C	N	Ar	Y_2_O_3_	Excess O
Balance	11.52	1.44	0.36	0.28	0.16	0.007	0.006	0.36	0.144

**Table 2:** Summary of coherent oxide dispersoid size and density for each irradiation case.

Specimen	Diameter (nm)	Density (Particles/m^3^)
Fe (350 nm)	2.3 ± 0.6	1.2 × 10^23^ ± 9.6 × 10^21^
He (350 nm)	2.6 ± 0.7	5.9 × 10^22^ ± 1.1 × 10^22^
1 × 10^15^ He + Fe (350 nm)	2.5 ± 0.5	1.6 × 10^23^ ± 1.2 × 10^22^
1 × 10^16^ He + Fe (350 nm)	2.6 ± 0.6	1.1 × 10^23^ ± 1.3 × 10^22^
Fe (2000 nm)	2.8 ± 0.6	6.0 × 10^22^ ± 4.0 × 10^21^
